# Supplementary figures and images for: Suspended multiwalled, acid-functionalized carbon nanotubes promote aggregation of the opportunistic pathogen Pseudomonas aeruginosa
Source: PLoS One. 2020 Jul 28;15(7):e0236599. doi: 10.1371/journal.pone.0236599 (PMC7386566; doi:10.1371/journal.pone.0236599)

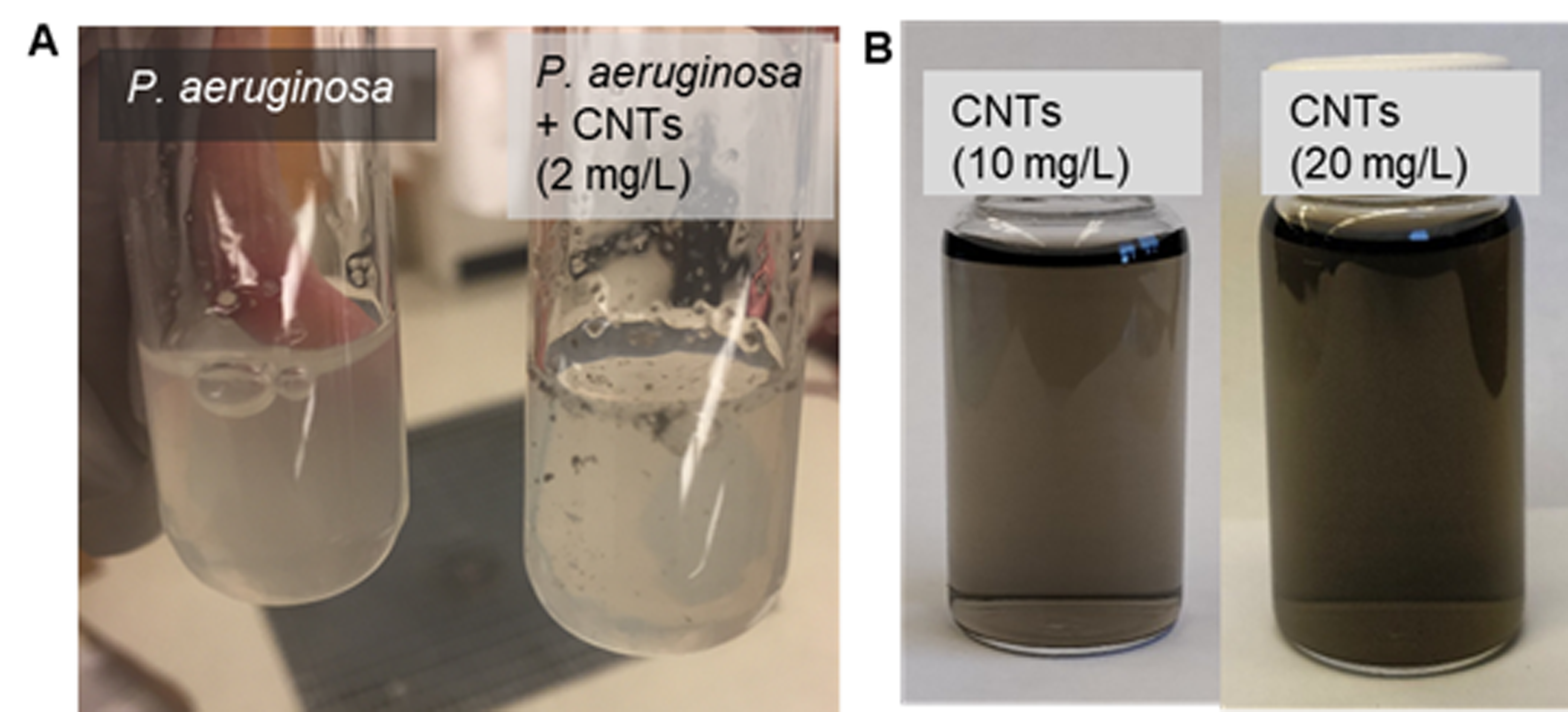

Supplement: S1 Fig — (A) When bacteria are grown in the presence of suspended MWCNTs, dark clumps appear in the culture after several hours of growth (right, P. aeruginosa + MWCNTs). Such clumps are not observed in a MWCNT-free culture grown under the same conditions (left, P. aeruginosa). (B) Because MWCNTs are functionalized with oxygenated groups (-OH, -COOH, -COH) to prevent aggregation, suspensions of MWCNTs without bacteria do not contain visible clumps even at high concentrations of MWCNTs. (TIF) [file pone.0236599.s001.tif]
